# Social participation of stroke patients: a bibliometric analysis

**DOI:** 10.3389/fneur.2025.1616861

**Published:** 2025-06-13

**Authors:** Jiaqing Yan, Yancheng Wang, Liang Zhang, Ping Chen, Nini Yang, Hongbo Zhang

**Affiliations:** ^1^Department of Nursing, Chongming Hospital Affiliated to Shanghai University of Medicine and Health Sciences, Shanghai, China; ^2^Graduate School, Shanghai University of Traditional Chinese Medicine, Shanghai, China

**Keywords:** stroke, social participation, WoS database, visual analysis, bibliometric

## Abstract

**Objective:**

Research on social functioning rehabilitation in stroke patients has received significant attention. In this study, we performed a bibliometric analysis using CiteSpace to examine publications focuses on post-stroke social participation between 2000 and 2025.

**Methods:**

Literature related to social participation of stroke patients was retrieved from the Web of Science Core Collection from January 1, 2000, to March 28, 2025, and the number of articles, countries, institutions, authors, references, and keywords were visualized and analyzed using Microsoft Office Excel and CiteSpace software.

**Results:**

The final analysis included836 publications, demonstrating a steady increase in annual publications over the 25-year period. Among contributing authors, Ng, Shamay S. M. demonstrated the highest productivity (20 publications). The United States and La Trobe University were the leading contributing countries and institutions. *“Archives of Physical Medicine and Rehabilitation”* was the most influential journal with a total of 600 citations (impact factor 3.6 in 2024). High-frequency keywords include “social participation,” “quality of life,” and “community integration.”

**Conclusion:**

This 25-year bibliometric analysis of post-stroke social participation research identifies priority areas for future studies.

## Introduction

The epidemiological burden of stroke, the second leading cause of mortality and third leading cause of disability worldwide, continues to rise (1). The Global Burden of Disease Study reports 12 million incident strokes annually worldwide, where 70–80% of survivors develop chronic functional impairments (1, 2). These impairments go beyond motor, cognitive, and linguistic deficits, severely limiting patients’ ability to perform daily activities and fulfill social roles. Notably, nearly 50% of stroke survivors still exhibit substantial participation problems1-year post-stroke, highlighting the progression from biological damage to limitations in social functioning (3, 4). In 2001, the World Health Organization introduced the International Classification of Functioning, Disability and Health (ICF), formally integrating societal-level participation as core metric for evaluating rehabilitation outcomes. This framework designated social participation as a research priority and a critical indicator of functional and prognostic recovery (5). Levasseur et al. defined social participation as a person’s involvement in activities that provide interaction with others in society or the community through analysis of 43 studies and expert consensus (6, 7).

Emerging evidence demonstrates significant associations between social participation levels and multidimensional health outcomes, including physical domains (motor function, communication deficits, cognitive impairments), psychological status (depression, motivation), and long-term quality of life. Crucially, social participation is recognized as the most robust independent predictor of rehabilitation outcomes (8, 9). Longitudinal studies indicate that low social participation is associated with elevated suPAR levels, a marker of chronic inflammation, suggesting a potential causal link. Elevated systemic inflammatory markers are linked to poor functional outcomes and increased mortality post-stroke (10–12). With the paradigm shift of the rehabilitation medicine model to a comprehensive “biopsychosocial” framework, the scope of social participation research has gradually expanded, and the relevant publications have demonstrated exponential growth (13). Given the growing recognition of social participation as a central outcome in stroke rehabilitation, it becomes crucial to understand how research in this area has evolved over time. However, existing studies focus on the current status, influencing factors, or intervention validation, while systematic analyses of knowledge architecture, disciplinary evolution, and international collaboration patterns remain understudied.

Traditional literature reviews primarily emphasize content analysis but often fail to identify emerging research hotspots and collaborative networks. Bibliometric analysis, conversely, is a quantitative methodology grounded in mathematics and statistics. This approach extracts metadata (authors, countries, institutions, keywords, cited references) from publications via analytical software, mapping a field’s macro-level landscape and effectively exploring its disciplinary evolution (14, 15). Bibliometrics has gain extensive application across medical disciplines, including complementary and alternative medicine (16), oncology (17), infectious diseases (18), nursing (19), and encephalopathy (20). This study employs bibliometric methods coupled with CiteSpace (version 6.2. R3), a scientometric visualization tool, to provide a panoramic analysis of global research dynamics in the field of stroke social participation, aiming to address current knowledge gaps. Through systematic examination of productive authors, institutional collaborations, keyword co-occurrences networks, and literature co-citation patterns, this study seeks to delineate the intellectual foundations, emerging hotspots, and frontier trends. These findings may guide evidence-based rehabilitation practices, policy design, interdisciplinary resource integration, and future research prioritization in stroke rehabilitation (21).

## Materials and methods

### Data collection and search strategy

This study follows a descriptive bibliometric design aimed at mapping research trends, collaboration patterns, and thematic evolution in the domain of stroke and social participation. Data were extracted from the Web of Science (WoS) Core Collection, a globally recognized and authoritative citation indexing database. As the world’s largest and oldest academic citation database, Web of Science encompasses rigorously vetted academic journals, review articles, conference proceedings, monographs, and other resources. Its core sub-collections include the Science Citation Index Expanded (SCI-EXPANDED), Current Chemical Reactions (CCR-EXPANDED), and Index Chemicus (IC) (22).

The literature search was performed on March 28, 2025. The search covered publications indexed in Web of Science from January 2000 to March 2025. Detailed search strings are provided in Supplementary Table 1.

Inclusion criteria:

Articles focused on stroke and social participation.
Published between January 2000 and March 2025.
Document type: original research articles and reviews.
Language: English.

Exclusion criteria:

Early access publications (*n* = 14).
Conference proceedings (*n* = 9).
Retracted publications (*n* = 1).

### Data extraction

Data collection was conducted by two nursing graduate students experienced in systematic reviews and meta-analyses. Two independent reviewers screened titles and abstracts to assess eligibility. Disagreements were resolved by consensus or through arbitration by a third reviewer. Cohen’s kappa coefficient could be calculated to assess inter-rater agreement, though it was not performed in this study. Preliminary document screening was executed through title or abstract assessment within the Web of Science platform and later exported in plain textual format containing the complete Web of Science record and cited references. Each record comprised author credential, publication title, periodical source, abstract, keywords, references, institution, funding, and citation information. Duplicate records were detected and removed using CiteSpace 6.2. R3 (23).

### Data analysis

Software tools:

CiteSpace 6.2. R3 and Microsoft Excel.

Analysis parameters:

Time slicing = 1 year, Timeframe = 2000–2025.

Network types:

Collaboration networks (authors, countries, institutions).
Co-citation networks (authors, journals, references).
Keyword co-occurrence and burst analysis.

Methodological details:

Microsoft Excel generated annual cumulative publication counts. CiteSpace 6.2. R3 (24) extracted three network types: collaboration, co-citation, and co-occurrence. In visualizations:

Node size corresponds to frequency.
Line thickness reflects co-occurrence strength.

Nodes with a centrality score greater than 0.1 are marked with a purple ring, indicating their pivotal role in connecting research subfields (25–27).

### Ethical considerations

As this study involved secondary analysis of bibliometric data from publicly available databases, no ethical approval was required. All procedures complied with Web of Science usage policies and academic integrity standards.

## Results

**Figure 1:**
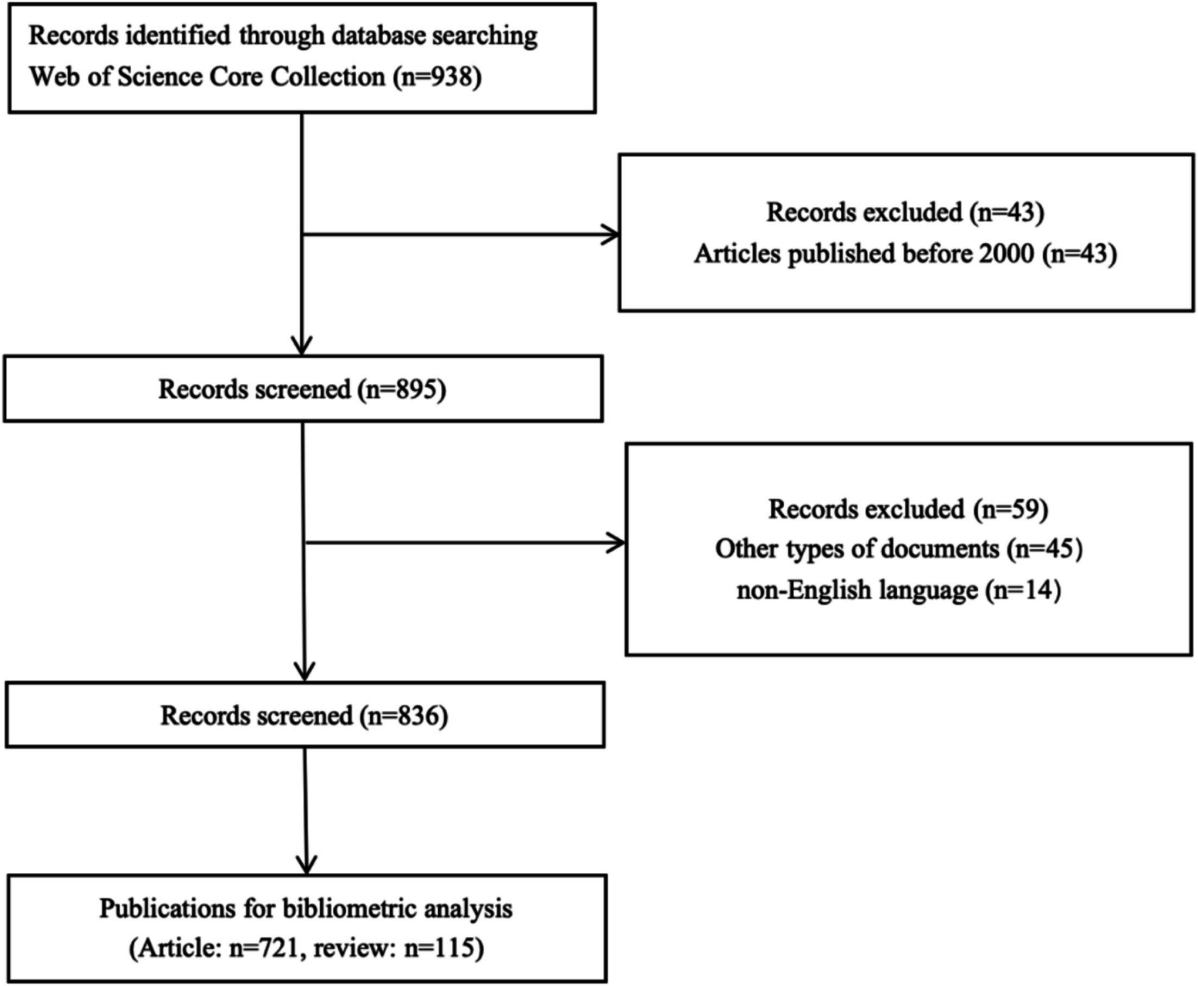
Flow diagram of study selection.

**Figure 2:**
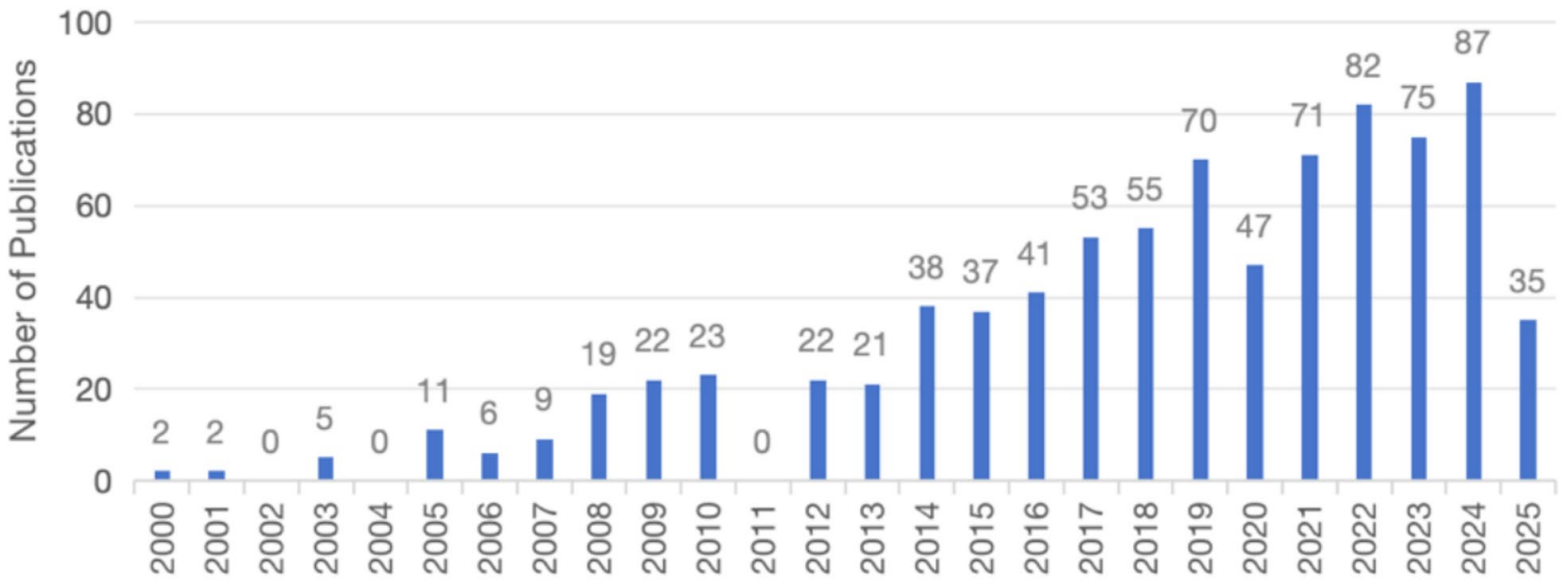
Annual publication trends in stroke social participation research (2000–2025).

Figure 1 illustrates the literature screening workflow. The initial search yielded 938 publications, with 836 meeting inclusion criteria. Figure 2 presents annual publication trends in stroke-related social participation from January 2000 to March 2025, revealing a sustained upward trajectory. Output peaked in 2024 (*n* = 87), representing a 44-fold increase from baseline levels in 2000 (*n* = 2). This exponential growth confirms sustained scholarly interest in the field and underscores a paradigm shift prioritizing social participation as a core target in stroke rehabilitation. These collective efforts provide a robust literature foundation for developing evidence-based community rehabilitation systems.

### Analysis of authors and co-cited authors

**Figure 3:**
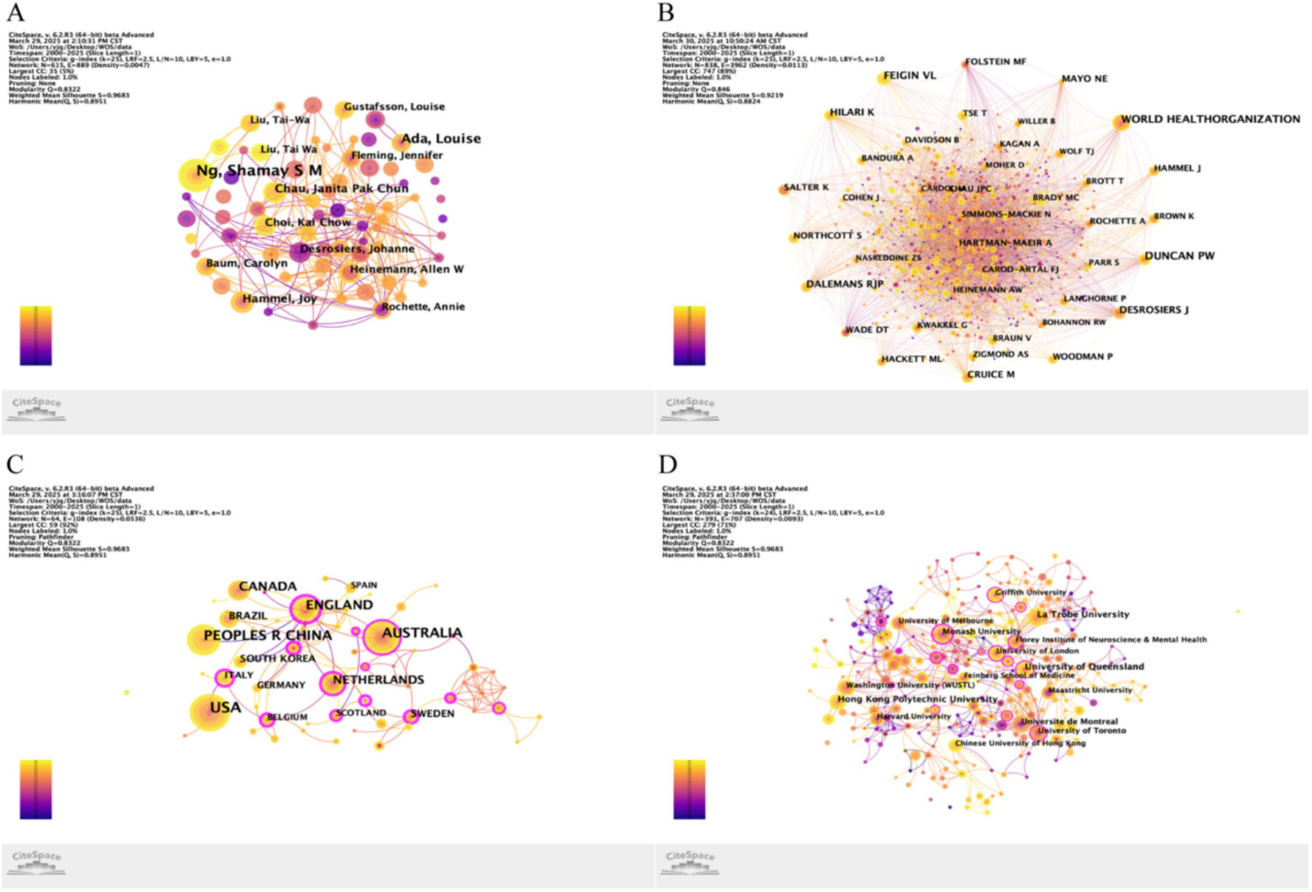
Multidimensional collaborative networks in stroke social participation research. **(A)** Author collaboration network. **(B)** Co-citation network of cited authors. **(C)** Country/region collaboration network. **(D)** Institutional collaboration network.

The analysis of 836 publications identified 3,637 unique authors. Figures 3A,B visualize author collaboration networks and co-citation networks, respectively. Table 1 ranks the top 10 authors and co-cited authors by publication volume. The most productive authors were Ng, Shamay S M (*n* = 20), Ada, Louise (*n* = 13), Hammel, Joy (*n* = 8), and Chau, Janita Pak Chun (*n* = 8), with Hammel, Joy and Chau, Janita Pak Chun tied for third position. These authors also exhibited strong collaborative ties. Co-cited author analysis identifies pivotal contributors through citation frequency. The top five co-cited authors were World Health Organization (*n* = 142), Duncan P. W. (*n* = 118), Feigin V. L. (*n* = 95), Desrosiers J. (*n* = 81), and Hilari K. (*n* = 78).

**Table 1:** Top 10 authors and co-cited authors by publication and citation frequency.

Rank	Author		Co-cited author	
	Sort by no. of articles published	Frequency	Sort by no. of cited articles	Citation
1	Ng, ShamayS M	20	World Health Organization	142
2	Ada, Louise	13	Duncan P. W.	118
3	Hammel, Joy	8	Feigin V. L.	95
4	Chau, Janita Pak Chun	8	Desrosiers J.	81
5	Desrosiers, Johanne	7	Hilari K.	78
6	Choi, Kai Chow	7	Dalemans R. J. P.	76
7	Heinemann, Allen W	7	Mayo N. E.	67
8	Baum, Carolyn	7	Cruice M.	61
9	Liu, Tai-Wa	6	Wade D. T.	49
10	Fleming, Jennifer	6	Hartman-maeir A.	47

### Analysis of countries and institutions

The 836 analyzed publications involved 3,075 institutions from 249 countries/regions. Figures 3C,D display country/regional and institutional collaboration networks, respectively. Table 2 ranks the top 10 countries/regions and institutions by publication volume. The United States (*n* = 199), Australia (*n* = 136), and China (*n* = 122) ranked as the top three contributors. Notably, the United States and Australia are classified as developed economies, whereas China is categorized as developing economy, indicating that dominant leadership by developed countries in steering disciplinary evolution. The top three countries/regions in terms of centrality were Wales (centrality = 0.61), Switzerland (0.56), and Sweden (0.55), which have critical roles in global collaboration.

**Table 2:** Top 10 countries/regions and institutions by publication output and centrality.

Rank	Countries/regions				Institutions			
	Sort by no. of articles published	Frequency	Sort by centrality	Centrality	Sort by no. of articles published	Frequency	Sort by centrality	Centrality
1	USA	199	Wales	0.61	La Trobe University	34	Monash University	0.49
2	Australia	136	Switzerland	0.56	Hong Kong Polytechnic University	34	University System of Ohio	0.3
3	Peoples R China	122	Sweden	0.55	University of Queensland	34	Florey Instite of Neuroscience &Mental Health	0.19
4	England	92	Belgium	0.45	Universite de Montreal	25	Case Western Reserve University	0.16
5	Canada	87	England	0.41	Monash University	25	University of London	0.15
6	Netherlands	65	Kenya	0.4	Washington University(WUSTL)	23	University of Queensland	0.14
7	Brazil	36	Scotland	0.31	University Of Toronto	21	Boston University	0.14
8	Sweden	28	Netherlands	0.3	University of Melbourne	20	Australian Catholic University	0.14
9	South Korea	26	Italy	0.23	University of London	20	Princess Alexandra Hospital	0.13
10	Italy	25	New Zealand	0.21	Griffith University	20	University of Ottawa	0.13

Among the top institutions by publication count, three were from Australia: La Trobe University (*n* = 34), the University of Queensland (*n* = 34), and Monash University (*n* = 25). Other leading institutions included Hong Kong Polytechnic University (*n* = 25; China), Université de Montréal (*n* = 25; Canada), and Washington University (*n* = 23; USA). CiteSpace’s betweenness centrality analysis identified Monash University (centrality = 0.49; Australia), the University System of Ohio (0.30; USA), and Florey Institute of Neuroscience and Mental Health (0.19; Australia) were the top 3 institutions. Monash University, a member of Australia’s Group of Eight, is recognized for its medical and engineering research. The centrality values of the top 3 institutions (all > 0.1) suggest strong collaborative connectivity, highlighting their pivotal roles in current research networks.

### Analysis of cited journals

**Figure 4:**
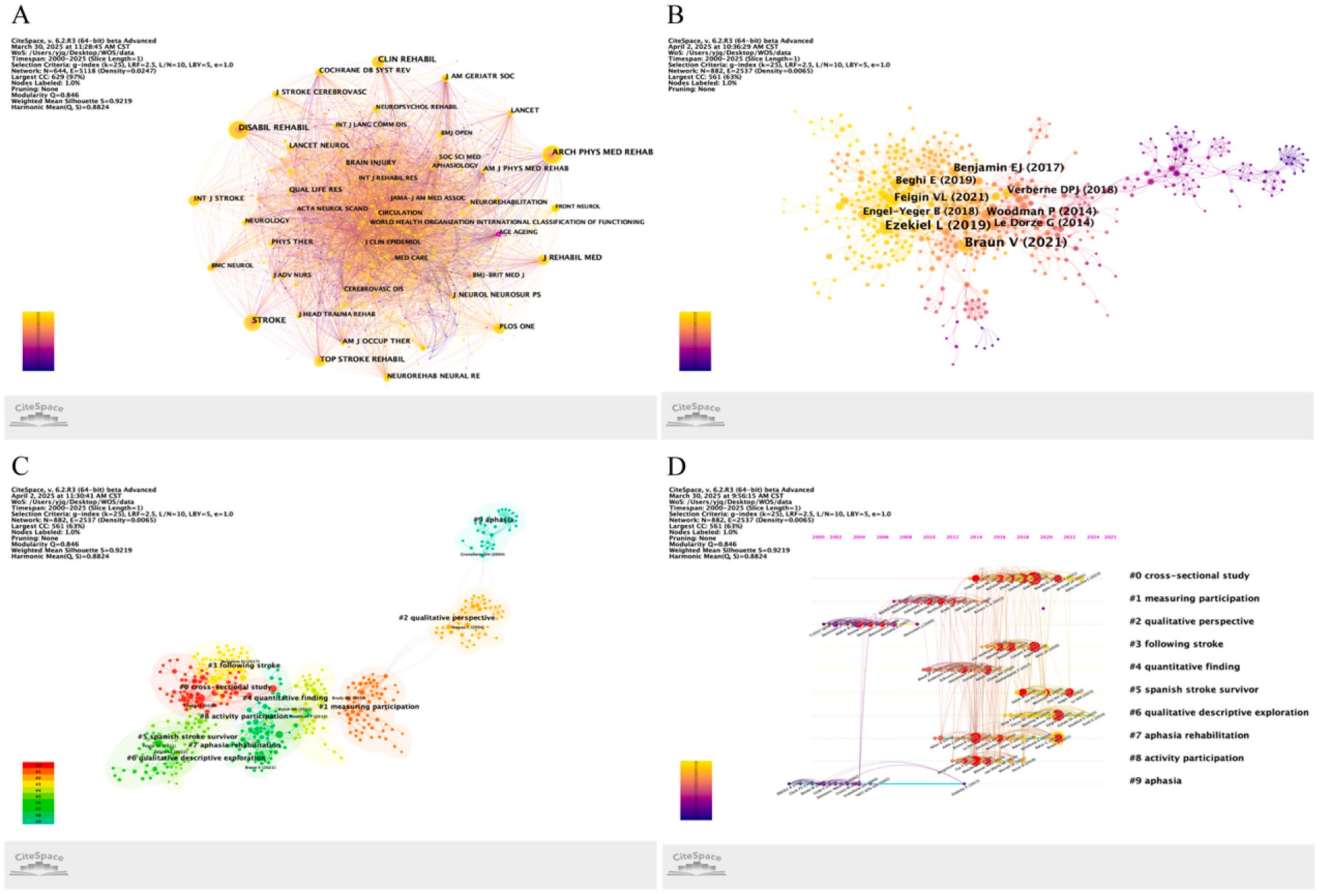
Bibliometric landscape of stroke social participation research on co-citation networks and temporal evolution. **(A)** Journal co-citation network. **(B)** Reference co-citation network. **(C)** Thematic cluster mapping of co-cited reference. **(D)** Timeline analysis of co-cited reference.

Figure 4A visualizes journal co-citation networks in stroke-related social participation research, with Table 3 ranking the top 10 cited journals. The most frequently cited journal were *Archives of Physical Medicine and Rehabilitation* (*n* = 600), *Disability and Rehabilitation* (*n* = 574) and *Stroke* (*n* = 554). The top 3 cited journals in terms of centrality were *Age & Ageing* (centrality = 0.11), *Social Science & Medicine* (0.08), *Brain Injury*, *Acta Psychiatrica Scandinavica*, *American Journal of Epidemiology*, and *Brain* (all 0.07).

**Table 3:** Top 10 cited journals by citation frequency and centrality.

Rank	Cited journals			
	Sort by no. of cited articles	Citation	Sort by centrality	Centrality
1	Arch Phys Med Rehab	600	Age Ageing	0.11
2	Disabil Rehabil	574	Soc Sci Med	0.08
3	Stroke	554	Brain Injury	0.07
4	Clin Rehabil	431	Acta Psychiat Scand	0.07
5	Top Stroke Rehabil	362	Am J Epidemiol	0.07
6	J Rehabil Med	335	Brain	0.07
7	Plos One	235	Arch Phys Med Rehab	0.06
8	Neurorehab Neural Re	226	Aphasiology	0.06
9	Int J Stroke	210	Aging Clin Exp Res	0.05
10	Lancet	208	J Am Geriatr Soc	0.05

### Analysis of cited reference

Figure 4B presents the reference co-citation network, with Table 4 ranking the top 10 cited references. The most frequently cited references were Braun V, 2021 (*n* = 31), Ezekiel L, 2019 (*n* = 26), Woodman P, 2014, and Feigin VL, 2021 (both *n* = 18). The top 3 references by betweenness centrality were Desrosiers J, 2008 (centrality = 0.21), Algurén B, 2012 (0.18) and Desrosiers J, 2005 (0.16). Figures 4C,D display cluster mapping and timeline analyses of co-cited references, revealing 15 thematic clusters. Major research domains include cross-sectional study, measuring participation, qualitative perspective, and following stroke. Supplementary Figure 1 delineated cutting-edge developments through citation bursts, identifying 40 most frequently cited references with prominent citation surges. The red bands denote burst duration, while blue stripes indicate the overall timeline.

**Table 4:** Top 10 cited references by citation frequency and centrality.

Rank	Cited references			
	Sort by no. of cited articles	Citation	Sort by centrality	Centrality
1	Braun V, 2021, Qual Res Psychol, V18, P328.	31	Desrosiers J, 2008, Neurorehab Neural Re. V22. P288.	0.21
2	Ezekiel L, 2019, Arch Phys Med Rehab, V100, P945.	26	Algurén B, 2012, Neurorehab Neural Re, V26, P266.	0.18
3	Woodman P, 2014, Disabil Rehabil, V36, P2031.	18	Desrosiers J, 2005, Jrehabil Med, V37. P353.	0.16
4	Feigin VL, 2021, Lancet Neurol, V20, P795.	18	Egan M, 2014, Arch Phys Med Rehab, V95, P262.	0.14
5	Benjamin EJ, 2017, Circulation, V135, PE146.	17	Feigin VL, 2014, Lancet, V383, P245.	0.1
6	Beghi E, 2019, Lancet Neurol, V18, P357.	15	Fotiadou D, 2014, Aphasiology, V28, P1281.	0.08
7	Le Dorze G, 2014, Aphasiology, V28, P421.	15	Graneheim UH, 2004, Nurs Educ Today, V24, P105.	0.08
8	Engel-Yeger B, 2018, Behav Neurol, V2018, PO.	14	COhen J., 2013, Statistical Power Analysis For The Behavioral Sciences, VO, PO	0.07
9	Verberne DPJ, 2018, Neurorehab Neural Re, V32, P821.	14	KOSSI D. 2018. Arch Phys Med Rehab. V99. P652.	0.07
10	Palstam A, 2019, Plos One, V14, PO. one.0219513	13	Ezekiel L, 2019, Arch Phys Med Rehab, V100, P945.	0.06

### Analysis of keywords

**Figure 5:**
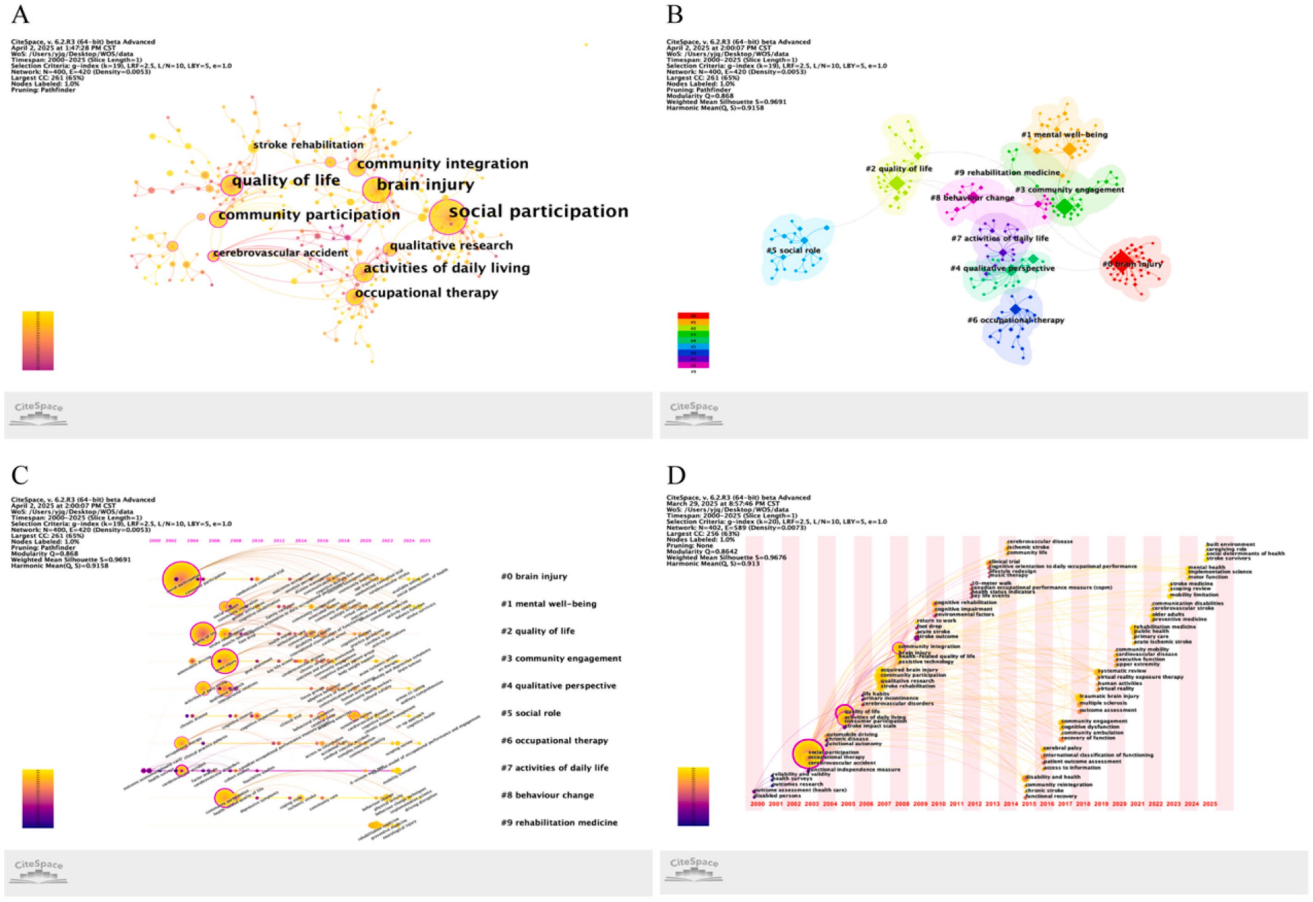
Bibliometric keyword analysis in stroke social participation research. **(A)** High-frequency keyword network. **(B)** Keyword cluster mapping. **(C)** Keyword timeline analysis. **(D)** Keyword time zone visualization.

Figure 5A visualizes high-frequency keywords in stroke-related social participation research, with Table 5 ranking the top 10 keywords by occurrence. The top 3 keywords by frequency were social participation (*n* = 138), quality of life (*n* = 66), and community integration (*n* = 47). The top 3 keywords by betweenness centrality were community participation (centrality = 0.41), cerebrovascular accident (0.33) and brain injury (0.27). Figures 5B,C display keyword cluster mapping and timeline analyses, identifying 15 thematic clusters focused on brain injury, mental well-being, and quality of life as emerging research frontiers. Figure 5D illustrates the keyword time zone visualization. Additionally, burst detection analysis identified 15 keywords exhibiting the strongest citation surges, with further details provided in Supplementary Figure 2.

**Table 5:** Top 10 keywords by frequency and centrality.

Rank	Keywords	Frequency	Keywords	Centrality
1	social participation	138	community participation	0.41
2	quality of life	66	cerebrovascular accident	0.33
3	community integration	47	brain injury	0.27
4	community participation	43	social participation	0.22
5	brain injury	37	quality of life	0.2
6	activities of daily living	36	acquired brain injury	0.2
7	occupational therapy	31	activities of daily living	0.15
8	acquired brain injury	26	community integration	0.12
9	qualitative research	22	qualitative research	0.12
10	traumatic brain injury	17	occupational therapy	0.11

## Discussion

This study conducted a bibliometric analysis of 836 publications on post-stroke social participation research spanning the period from 2000 to 2025. Data were retrieved from the Web of Science Core Collection and analyzed using CiteSpace for network visualization. This study provides a comprehensive bibliometric overview of the field, representing one of the earliest efforts to systematize publication trends and research dynamics on social participation post-stroke. By identifying productive authors, key institutions, leading countries/regions, collaboration networks, and core journals, this study provides critical references for researchers and practitioners. The visual analytics revealed emerging trends and research gaps in post-stroke social participation, while delineating critical knowledge domains to inform future investigations. Analysis demonstrated steady growth from 2000 to 2025, peaking in 2024 with 87 annual publications.

### Synergistic interactions and network analysis

Co-authorship network analysis showed that 3,637 scholars have contributed to stroke social participation research, demonstrating relatively strong collaborative linkages. Enhancing domestic institutional collaboration could accelerate stroke social participation research through multidisciplinary integration (neurology, rehabilitation engineering, psychology, and public health), enabling nationwide monitoring of social functioning dynamics and standardized interventions while advancing culturally contextualized community integration strategies. Conversely, fostering international partnerships enables comparative analyses of sociocultural barriers to post-stroke social participation and evidence-based evaluation of rehabilitation models across healthcare systems. Deepening global collaboration could integrate cross-regional research approaches (e.g., context-specific digital health applications in high-resource versus low-resource regions) and rely on cross-national cohort studies (28) (e.g., the ENOS project) to address complex challenges such as vocational and social reintegration-goals requiring multilateral synergies beyond the capacity of isolated teams or regions. The study identifies core contributors including Ng, Shamay S. M., Ada, Louise, Hammel, Joy, and Chau, Janita Pak Chun, whose foundational works define the disciplinary framework.

### Country/regional and institutional analysis

This study identified the United States as the global leader in multinational collaborations and academic productivity in stroke social participation research. This dominance likely stems from sustained investments in neurorehabilitation, including dedicated NIH funding (29), and mature clinic-academic partnerships exemplified by Washington University and Northwestern University’s digital self-management interventions for stroke patient self-management, relying on clinical resources at hospitals such as Barnes-Jewish (30). At the institutional level, Australia demonstrated concentrated research capacity, with La Trobe University, University of Queensland, and Monash University ranking top 3 globally in publications output, which may be related to the Australian government’s funding of stroke social functioning reconstruction initiatives and its *Australian and New Zealand Living Clinical Guidelines for Stroke Management*, which mandate that “stroke rehabilitation must commence on day one as integrated process to maximize social participation” (31). Nevertheless, global collaboration for stroke research remains fragmented, with 51 out of 64 countries and 373 out of 391 institutions having a centrality value of less than 0.1. This underlines the fragmented and potentially siloed nature of efforts, which may reduce the replicability and scalability of interventions across sociocultural contexts (32). It is also worth noting the relative underrepresentation of low- and middle-income countries in the global collaboration network, which could mask region-specific needs and hinder inclusive knowledge generation. To bridge this gap, establishing a collaborative framework that strategically utilizes institutional resource is imperative. Accelerating global optimization of social participation assessment tools and rehabilitation programs through multi-center cohort data sharing and culturally adaptive intervention design could effectively translate evidence into practice.

### Cited journals and cited literature analysis

Journal co-citation analysis identified *Archives of Physical Medicine and Rehabilitation* as the most frequently cited journal. Researchers should prioritize submission to these journals and systematically review existing literature. Reference co-citation analysis revealed four core research themes: cross-sectional studies, participation measurement, qualitative perspectives, and post-stroke. Among these, studies on aphasia rehabilitation, cross-sectional studies, quantitative studies, and qualitative descriptive studies received the highest citation frequency, highlighting aphasia rehabilitation not only as a dominant thematic focus but also as a representative challenge that bridges neurological, psychological, and communicative dimensions in post-stroke recovery (33). Consequently, current research priorities are shifting toward supporting social participation in post-stroke aphasia, transcending impairment-centric rehabilitation models. These highly cited works represent key research findings in the construction of a body of knowledge in this area.

### Keyword analysis

Keyword co-occurrence analysis identified “social participation” “quality of life” and “community integration” as core themes in stroke rehabilitation research from 2000 to 2025 reflecting t sustained focus on functional recovery and social role reconstruction. Burst detection analysis revealed that research priorities over this period centered on traditional dimensions such as outcome assessment (health care) disabled persons and cerebrovascular accident indicating a persistent emphasis on quantitative assessment and disease ontology. These findings highlight opportunities for future researchers to explore underexamined areas to expand the literature landscape (34). Cluster analysis revealed “brain injury” as a core research cluster consistent with its pathophysiological-functional relevance. Stroke as an acute cerebrovascular event is essentially a typical type of acquired brain injury caused by focal cerebral ischemia or hemorrhage. This clustering suggests that studies on post-stroke social participation barriers predominantly emphasize neurological deficits triggered by brain injury subtypes and their impact on social role reintegration (35). The prominence of brain injury may stem from two factors: First motor dysfunction cognitive deficits and communication deficits caused by brain injury together constitute the fundamental barriers to patients’ reintegration into social roles and right hemisphere injury is particularly associated with impairments in key social interaction abilities such as affective recognition deficits and discourse communication deficits (35, 36). Second brain injury serves as an overarching conceptual framework with its assessment tools and intervention strategies widely adapted in stroke social participation research-a methodological cross-pollination driving bibliometric clustering. The “Mentalwell-being” cluster focuses on post-stroke psychosocial adaptation deficits and their dynamic impacts on social participation. A case–control study comparing 51 brain injury survivors with 51 matched healthy controls revealed significantly elevated loneliness depressive symptoms diminished quality of life and emotional well-being in the patient cohort (37). The “quality of life” cluster emphasizes the multidimensional impact of social participation on post-stroke well-being. Stroke-related disabilities impair both physical functioning and social engagement with prior studies documenting substantial declines in survivors’ quality of life (38). The keyword and cluster analyses reinforce the findings from the network analysis and country-level evaluation highlighting a shared focus on quality of life community integration and the multidimensional challenges of post-stroke reintegration. In summary future research should integrate transdiagnostic frameworks implement psychosocial and mental health interventions and develop personalized strategies with multidimensional quality of life assessment to facilitate the reconstruction of patients’ social roles and functional recovery.

### Strengths, limitations, and future directions

This study provides a comprehensive bibliometric overview of social participation in stroke patients, systematically mapping emerging research trends, shifts in global research priorities, and key thematic areas, thereby establishing a foundational framework to advance scholarly discourse in this field. However, three limitations warrant consideration: first, data were exclusively sourced from the Web of Science database, potentially limiting comprehensiveness; second, the study focused on Articles and Reviews, which may lead to the omission of some of the important themes; third, including only English-language publications may have excluded culturally specific perspectives and non-Western rehabilitation models, thereby limiting the inclusivity and contextual applicability of the insights generated. Future studies could enhance coverage by integrating multiple databases. Expanding search strategies to incorporate alternative terminology and non-English literature would further refine analyses and better capture global trends in social participation of stroke patients.

## Conclusion

This bibliometric analysis systematically mapped advancements and trends in stroke social participation research from 2000 to 2025. The findings demonstrate sustained growth in publications output, with the United States and Australia dominant research productivity. *Archives of Physical Medicine and Rehabilitation* emerged as the most frequently cited journal, while cross-sectional study, measuring participation, qualitative perspectives, and following stroke constituted core research themes. The highest citation frequencies were observed in studies on aphasia rehabilitation, cross-sectional study, quantitative finding, and qualitative descriptive exploration. High-frequency keywords comprised “social participation,” “quality of life,” and “community integration,” whereas the strongest citation bursts centered on outcome assessment (health care), disabled persons, cerebrovascular accident. These findings provide researchers with critical insights to identify collaborative networks and contextualize the current literature landscape, directly informing innovation in post-stroke rehabilitation strategies.

## Data Availability

The datasets presented in this study can be found in online repositories. The names of the repository/repositories and accession number(s) can be found in the article/Supplementary material.
