# Supplementary material for: Social participation of stroke patients: a bibliometric analysis
Source: Front Neurol. 2025 Jun 13;16:1616861. doi: 10.3389/fneur.2025.1616861 (PMC12202427; doi:10.3389/fneur.2025.1616861)
Supplement: Supplementary file 1 [file Data_Sheet_1.pdf]

Table S1. Details of search strategy

Number	Search term
1	TS= (Stroke)
2	TS= (Apoplexy)
3	TS= (Cerebrovascular Accident)
4	TS= (CVA)
5	TS= (Cerebral Infarction)
6	TS= (Intracranial Hemorrhage)
7	TS= (Social Participation)
8	TS= (Social Engagement)
9	TS= (Social Involvement)
10	TS= (Social Integration)
11	TS= (Community Participation)
12	TS= (Community Engagement)
13	TS= (Community Engagement)
14	TS= (Community Involvement)
15	TS= (Community Integration)
16	1 OR 2 OR 3 OR 4 OR 5 OR 6
17	7 OR 8 OR 9 OR 10 OR 11 OR 12 OR 13 OR 14 OR 15
3	16 AND 17

TS=title, abstract, author keywords and keywords plus.

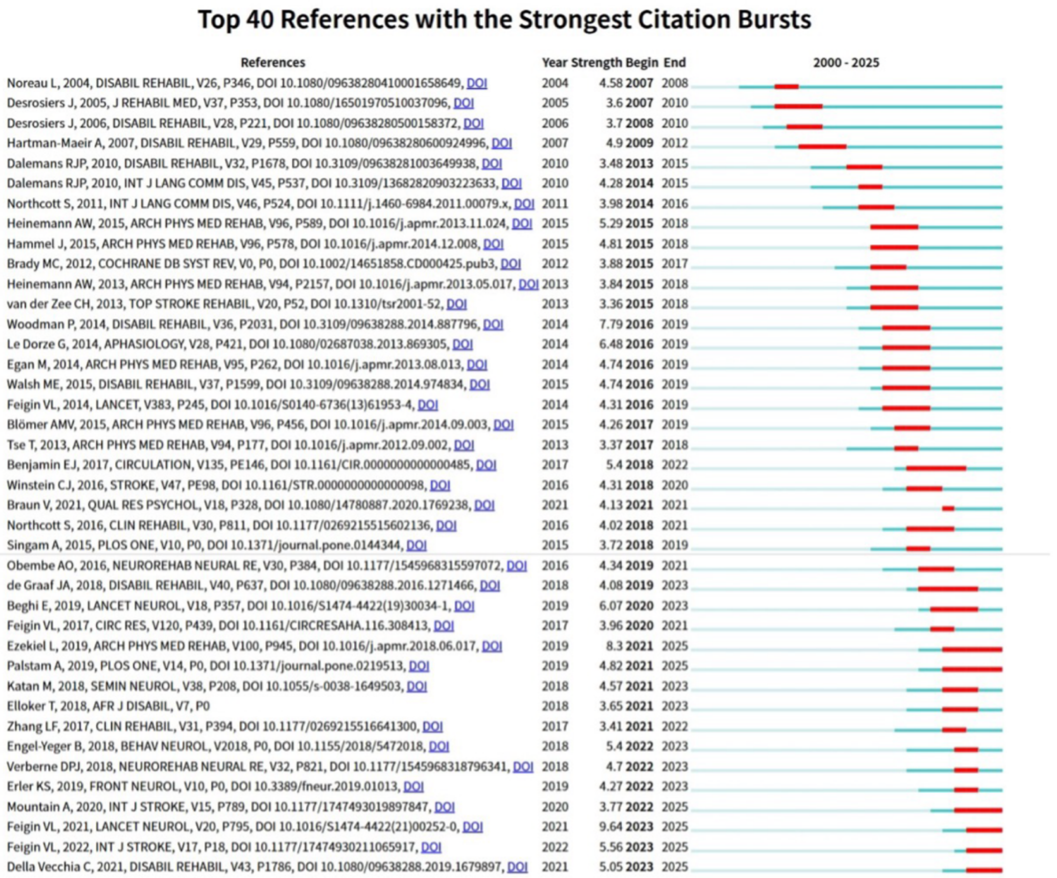

Figure S1 Burst Detection of Co-cited References

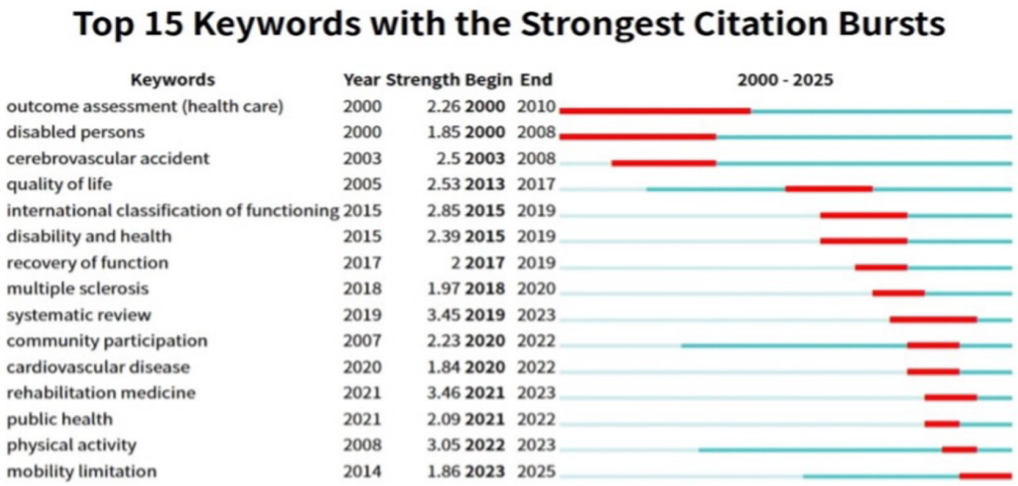

FigureS2 Burst Detection of Keywords
